# Book Review

**Published:** 1904-03

**Authors:** 


**Surgical Bandaging and Dressings.** By Wm. Johnson Smith. Pp. viii., 167. London: The Scientific Press Ltd.—A useful little pocket-book for dressers and nurses, explaining the principles of aseptic and antiseptic surgery, the treatment of wounds and ulcers, and the application of bandages, splints and strappings. The latter part is profusely and well illustrated, but the splint pictured as Carr's for fractured radius is not the one usually understood as "Carr's."

**Modern Methods in the Surgery of Paralyses.** By A. H. Tubby and Robert Jones. Pp. xiii., 311. London: Macmillan & Co., Ltd. 1903.—By means of muscle-grafting, tendon-transplantation and arthrodesis, modern surgery has done much to mitigate and remedy the helpless and hopeless condition of many patients afflicted with an incurable paralysis, and the present volume is a timely exposition of the means by which so much good has been accomplished. Nearly two-thirds of the book is taken up with infantile paralysis, and the methods by which the various disablements and deformities which arise in this condition can be remedied are duly and lucidly discussed. In some of the illustrative cases the results are most gratifying and almost astonishing, and yet the manner of accomplishment is in most cases simple enough. It is doubtful whether any good purpose is served by a brief discussion of the ætiology and pathology of this condition in a work which deals mainly with the surgical side of the matter; and it is, perhaps, not in the best taste to state how various patients made "endless journeys" to "surgeons and physicians of repute," and consulted the "best of our neurologists" without benefit before coming under the care of the authors. It is very properly pointed out that the recognition of the differences between the disabilities due to mechanical causes and those due to central pathological changes will cause much of the difficulty in treatment to disappear. The second section of the book deals with infantile spastic paralysis, and the third section with various paralyses and deformities arising from injuries and diseases of nerves and some degenerations of the spinal cord. The underlying principles of treatment are the same in all, and the descriptions of the various methods are clear and concise and freely illustrated by helpful illustrations. The book can be safely recommended to all who are interested in orthopædic surgery.

**Modern Materia Medica and Therapeutics.** By A. A. Stevens, M.D. Third Edition. Pp. 663. Philadelphia: W. B. Saunders and Company. 1903.—This edition has been re-arranged, inasmuch as the drugs are no longer in alphabetic order but are classified according to their pharmacologic action. All the well-known drugs are reviewed briefly and without the introduction of controversial topics. It is interesting to note that amongst circulatory stimulants is included the extract of suprarenal capsule (p. 58), and the physiologic and therapeutic actions of the suprarenal glands are described at greater length (p. 338). As a local vaso-constrictor adrenalin has proved to be exceedingly valuable, but for internal medication its utility is not yet firmly established. Extravagant statements are being made, which have yet to be proved. A writer in the *Medical Examiner* (Sept., 1903, p. 536) expresses his belief that "if a man's emunctories are in very good condition when he reaches the age of sixty-five, there is no doubt in my mind that he can live to one hundred, or longer, if he be furnished with the adrenal secretion." It has yet to be proved that suprarenal extract is a useful remedy in Addison's disease. Another organ which is being endowed with magic properties is the pituitary body: this, according to Sajous, is a very important part of the adrenal system, and controls not only the circulatory system, but is a governing centre of the nervous system also. This book gives very little evidence either of one or the other. The author believes that even with the extract of the testicle of rabbits, by which Brown-Séquard produced such remarkable effects of rejuvenation, in no instance has the good achieved been so decided as to convince a judicious observer that it was not due to mental suggestion rather than to the physiologic activity of the drug itself. Amongst the antipyretic drugs it is stated that phenacetin is the most satisfactory of the coal-tar series, and when used in moderate doses it is rarely followed by cyanois, collapse, cutaneous eruptions, and other untoward effect. This is in accord with general experience, and phenacetin has accordingly almost entirely displaced all other antipyretics. The chapters on applied therapeutics contain the usual summary of the treatment of various diseases. There is little which is new, but the author is a safe guide who admits nothing but that which is established on a satisfactory basis.

